# Can Natural Polyphenols Help in Reducing Cytokine Storm in COVID-19 Patients?

**DOI:** 10.3390/molecules25245888

**Published:** 2020-12-12

**Authors:** Giovanna Giovinazzo, Carmela Gerardi, Caterina Uberti-Foppa, Lucia Lopalco

**Affiliations:** 1 CNR-ISPA, Institute of Science of Food Production, National Research Council, 73100 Lecce, Italy; carmela.gerardi@ispa.cnr.it; 2 San Raffaele Scientific Institute, Vita-Salute University, 20132 Milan, Italy; uberti.caterina@hsr.it; 3 Division Immunology, Transplantation and Infectious Diseases, San Raffaele Scientific Institute, 20132 Milan, Italy

**Keywords:** polyphenols, SARS-CoV-2, COVID-19, cytokines, inflammation

## Abstract

SARS-CoV-2 first emerged in China during late 2019 and rapidly spread all over the world. Alterations in the inflammatory cytokines pathway represent a strong signature during SARS-COV-2 infection and correlate with poor prognosis and severity of the illness. The hyper-activation of the immune system results in an acute severe systemic inflammatory response named cytokine release syndrome (CRS). No effective prophylactic or post-exposure treatments are available, although some anti-inflammatory compounds are currently in clinical trials. Studies of plant extracts and natural compounds show that polyphenols can play a beneficial role in the prevention and the progress of chronic diseases related to inflammation. The aim of this manuscript is to review the published background on the possible effectiveness of polyphenols to fight SARS-COV-2 infection, contributing to the reduction of inflammation. Here, some of the anti-inflammatory therapies are discussed and although great progress has been made though this year, there is no proven cytokine blocking agents for COVID currently used in clinical practice. In this regard, bioactive phytochemicals such as polyphenols may become promising tools to be used as adjuvants in the treatment of SARS-CoV-2 infection. Such nutrients, with anti-inflammatory and antioxidant properties, associated to classical anti-inflammatory drugs, could help in reducing the inflammation in patients with COVID-19.

## 1. Introduction

The emerging severe acute respiratory syndrome (SARS)-coronavirus (CoV-2) is a novel airborne coronavirus threatening public health that is currently spreading worldwide, causing the severe respiratory syndrome known as COVID-19 [1]. This new pandemic emerged in Wuhan City, Hubei province of China, during late in 2019, resulting in a rapid spread all over the world [2].

The COVID-19 outbreak situation on 28 November 2020 was 61,036,793 confirmed cases of SARS-COV-2 infection with 1,433,316 deaths, as reported by the World Health Organization (WHO) [3].

One of the crucial questions regarding the current COVID-19 pandemic is the broad spectrum of disease severity ranging from mild to critical. Usual clinical symptoms of COVID-19 are fever, cough, short breath, asthenia, ageusia, anosmia, headache, myalgia, diarrhea, and confusion. Data from China showed that a fraction of patients developed severe illness requiring hospital care, including severe pneumonia, which can result in an acute respiratory distress syndrome (ARDS), with a possible dysfunction of several organs and, in some cases, death. Although patients with severe COVID-19 have lymphcytopenia, the lymphocytes are highly activated and result in a decrease of both CD4 and CD8 T cells, with functional exhaustion of cytotoxic T lymphocytes and a marked reduction of NK cells. All these immune dysfunctions induce an important reduction of antiviral protective immune responses, and play a relevant role in the pathogenesis and severity of COVID-19 [4]. Moreover, COVID-19 severity and hyper-inflammatory disorders share similarities concerning the onset of a cytokine storm linked to the histiocytic, reticulo-endothelial (monocytes/macrophages) system.

## 2. Cytokine Storm

The majority of patients with severe COVID-19 experienced an high level of pro-inflammatory response resulting in the CRS with a striking increased level of several pro- and anti-inflammatory cytokines and chemokines, such as interferon (IFN)-γ, interferon gamma induced protein 10 (IP-10), interleukin (IL)-1ra, IL-2ra, IL-6, IL-10, IL-18, hepatocyte growth factor (HGF), monocyte chemotactic protein-3 (MCP-3), macrophage colony stimulating factor (M-CSF), granulocyte colony-stimulating factor (G-CSF), monokine induced gamma interferon (MIG), macrophage inflammatory protein 1 alpha (MIG-1a), RANTES, CCL2, CCL3, and cutaneous T-cell-attracting chemokine (CTACK [5,6].

IP-10, MCP-3, and IL-1ra were strongly associated with severe cases of the illness [7], suggesting that the excess and aberrant level of inflammatory cytokine is extremely relevant during the progression of the COVID-19 disease. Indeed, it has been found that the abnormal and sustained levels of cytokines correlate with the lung damage and lethal effect in patients with COVID-19. Moreover, the abnormal cytokine release has been investigated at transcriptomic level as well and a sustained pro-inflammatory cytokine pathway has been found in both bronco alveolar lavage (BAL) and PBMC from COVID-19 patients. In detail, high levels of CXCL1, CXCL2, CXCL6, CXCL8, CXCL10/IP-10, CCL2/MCP-1, CCL3/MIP-1A, CCL4/MIP-1B, CCL8, IL33, and CCL3L1 have been detected in BAL samples, whereas CXCL10, TNFSF10, TIMP1, C5, IL18, AREG, NRG1, and IL-10 have been revealed in PBMC. Taken together, these transcriptional changes suggest a critical role played by the cytokine network in COVID-19 pathogenicity [7]. The pro-inflammatory effect of IL-6, which is a multifunctional cytokine, exerts its function in the modulation of the inflammatory storm. The major functions of IL6 are the T cell activation and promotion of B differentiation, with a consequent production of antibodies [8].

## 3. Potential Anti-Inflammatory Compounds against SARS-CoV-2

Cytokine storm is relatively common in severe cases of COVID-19. Several immunosuppressive therapies aimed at limiting immune-mediate damage due to cytokine storm are at various phases of development. Cytokine blocking agents are effective treatments after chimeric antigen receptor T-cell treatment (CAR-T), and that constituted the rational for the use of these drugs in patients with severe COVID-19 [9,10].

Interleukin 6 was reported to be increased in SARS and MERS patients and might play a role in the pathogenesis of these diseases [11,12]. The IL-6 antagonist tocilizumab has been shown to be effective against cytokine release syndrome, resulting from CAR-T cell infusion against B cell acute lymphoblastic leukemia. Thus, tocilizumab can be used to treat severe COVID-19 [13,14]; indeed, observational studies have shown promising results [15,16].

A large retrospective cohort study has been conducted on 544 patients with severe SARS-CoV-2 pneumonia. The criteria used to prescribe tocilizumab were oxygen saturation <92% in room air and a PaO_2_/FiO_2_ < 250 mmHg or a decrease in PaO_2_/FiO_2_ greater than 30% in the last 24 h. The risk of death/invasive mechanical ventilation was reduced in participants treated with tocilizumab, and the impact on 14-day mortality was greater, with 73 (20%) patients in the standard care group, compared with 13 (7%; *p* < 0.001) patients treated with tocilizumab [17]. Encouraging results were also obtained in patients with severe pneumonia in two cohorts of COVID-19 critical patients admitted to the intensive care unit [15,16]. These results were obtained in patients with severe pneumonia, but the outcomes of therapy may differ in different clinical pictures.

A press release from the Italian Medicine Agency (AIFA) indicated that a randomized multicentre study comparing tocilizumab to standard of care showed that there was no difference between the two arms and was concluded early [18]. In this case, tocilizumab was administered at an early stage, with recent onset requiring hospital care, but not invasive or semi-invasive mechanical ventilation procedures.

A systematic review and meta-analysis conducted by Zhao at al. demonstrated the efficacy of tocilizumab treatment in severely ill patients with COVID-19, despite the limitations due to the retrospective design of the studies evaluated [19].

Recently, a Roche press release stated that a Phase III double-blind, randomized trial on the safety and efficacy of tocilizumab, in patients with severe pneumonia, did not meet its primary endpoint of improved clinical status in hospitalized adult patients with severe COVID-19-associated pneumonia. In addition, the key secondary endpoints, which included the difference in patient mortality at week four, were not met [20].

A recent press release from Sanofi stated that sarilumab, another IL-6 antagonist failed to meet the primary endpoint in a randomized trial. The primary analysis group included 194 patients who were critically ill with COVID-19 and receiving mechanical ventilation at the time of enrolment [21].

Recently, in a retrospective cohort study of patients with COVID-19 and ARDS managed with non-invasive ventilation outside of the ICU, treatment with high-dose anakinra, a recombinant interleukin-1 receptor antagonist, was safe and associated with clinical improvement in 72% of patients [22]. The confirmation of its efficacy will require controlled trials.

Regarding adverse events of immunomodulatory drugs, tocilizumab was associated with an increased risk of infectious complications when compared to the standard of care. In particular, in the paper of Guaraldi et al., 24 (13%) of 179 patients treated with tocilizumab were diagnosed with new other infections versus 14 (4%) of 365 patients treated with the standard of care alone (*p* < 0.001) [17]. This finding is in contrast with those among patients undergoing CAR- (Chimeric Antigen Receptor) T cells therapy, who did not show an increase risk of infections [23].

The few patients treated with anakinra were evaluated only for the risk of developing bacteremia and there was no difference when compared to patients receiving standard treatment [22].

Janus kinase (JAK) inhibitors have been also suggested as drugs to treat COVID-19 due to their anti-inflammatory activity [24,25]. The most interesting drug of this group is baricitinib. Preliminary results on baricitinib plus corticosteroids, compared to corticosteroids alone, was associated with improved pulmonary function in patients with moderate to severe COVID-19 pneumonia [25]. Until now, all international guidelines agree to recommend immunomodulatory drug use in COVID-19, but only in the context of clinical trials [26].

Among anti-inflammatory drugs, glucocorticoids have an important role. At the beginning of the epidemic, the use of these drugs was somehow discouraged. Until August 2020, the WHO recommended against the routine use of corticosteroids in patients with COVID-19 for treatment of viral pneumonia or ARDS, unless indicated for another reason. The RECOVERY Trial conducted in the UK changed this position [27,28]. The trial showed that dexamethasone, at the dose of 6 mg given once daily for up to ten days compared to standard of care, reduced deaths by one-third in patients receiving invasive mechanical ventilation (29.0% vs. 40.7%, RR 0.65 [95% CI 0.51 to 0.82]; *p* < 0.001), by one-fifth in patients receiving oxygen without invasive mechanical ventilation (21.5% vs. 25.0%, RR 0.80 [95% CI 0.70 to 0.92]; *p* = 0.002), but did not reduce mortality in patients not receiving respiratory support at randomization (17.0% vs. 13.2%, RR 1.22 [95% CI 0.93 to 1.61]; *p* = 0.14). To date, on the basis of these results and other data from RCTs evaluating systemic corticosteroids versus usual care in COVID-19, all international guidelines strongly suggest the use of systemic (i.e., intravenous or oral) corticosteroid therapy (e.g., 6 mg of dexamethasone orally or intravenously daily or 50 mg of hydrocortisone intravenously every 8 h) for 7 to 10 days in patients with severe and critical COVID-19, while recommending not to use corticosteroid therapy in subjects not receiving respiratory support [29,30].

The main concern is that anti-inflammatory medications, such as corticosteroid, may delay the elimination of the virus and increase the risk of secondary infection, especially in those with an impaired immune system. Another consideration is that biological agents targeting pro-inflammatory cytokines can only inhibit specific inflammatory factor, and thus may not be very effective in the control the cytokine storm. The awareness of the “current knowledge gap” helps us to balance the risk and benefit ratio in the application of anti-inflammation therapy to patients with COVID-19.

## 4. Polyphenols as Natural Molecules with Anti-Inflammatory Activity 

**Figure 1:**
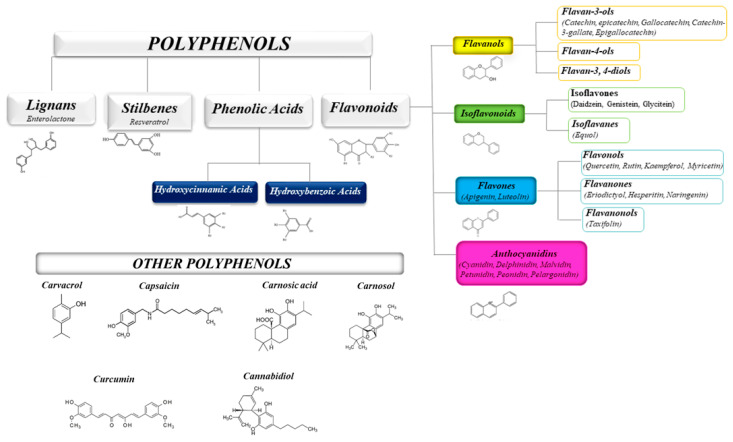
Schematic classification of the polyphenol family and flavonoid groups putatively exploited as antiviral phytochemical.

The health-promoting activities of plant polyphenols have been widely ascertained by numerous scientific publications. Studies on plant extracts and phytochemicals showed that polyphenols can play an anti-inflammatory action in the prevention and the progression of chronic diseases [31,32,33,34,35]. Polyphenols are ubiquitous in plants, being the products of plants’ secondary metabolism present either as glycosides or as free aglycones [36] (Figure 1). Thousands of structural variants (more than 8000) exist in the polyphenol family. In the plant kingdom, polyphenols determinate color, flavor, and defense activity [32]. They are grouped according to chemical structures into flavonoids such as flavones, flavonols, flavanol isoflavones, anthocyanins, proanthocyanidins, and non flavonoids, such as phenolic acids, and stilbenes [37,38]. Nowadays, the consumers prefer using natural food ingredients due to their plethora of healthy properties [39,40].

The anti-inflammatory activity of plant polyphenols has been demonstrated by in vitro and in vivo studies supporting their role as therapeutic tools in different acute and chronic disorders [35,41,42].

The ability of polyphenols to modulate the expression of several pro-inflammatory genes as well as the immune system, in addition to their anti-oxidant activity, contributes to the regulation of inflammatory signaling [43,44,45] (see Table 1).

Indeed, resveratrol from red wine showed an anti-atherogenesis property mainly due to its anti-inflammatory properties. In vivo and in vitro studies (in murine and rat macrophages) showed that resveratrol inhibited COX, peroxisome proliferator-activated receptor gamma (PPARγ), and activated eNOS (endothelial nitric oxide synthase) [54,56,57,62]. Curcumin and its chemical analogues were shown to inhibit NF-κB activated by several different inflammatory stimuli [51,63]. Moreover, curcumin and analogues reduced the expression of inflammatory cytokines: tumor necrosis factor (TNF) and IL-1, adhesion molecules like intercellular adhesion molecule-1 (ICAM-1), and vascular cell adhesion molecule-1 (VCAM-1) in human cell systems.

Macrophages are affected by polyphenols as well. Macrophages initiate inflammation by secreting pro-inflammatory mediators and cytokines like IL-6 and TNF-α. Polyphenols such as ferulic acid and coumaric acid (from propolis) reduce the production of TNF-α, interleukine-1-beta (IL-1-β) and IL-6 expression by inhibiting cyclooxygenase-2 (COX-2), and inducible nitric oxide synthase (iNOS) [52,53,58,64,65]. 

### 4.1. Polyphenols and Cytokine Modulation 

**Figure 2:**
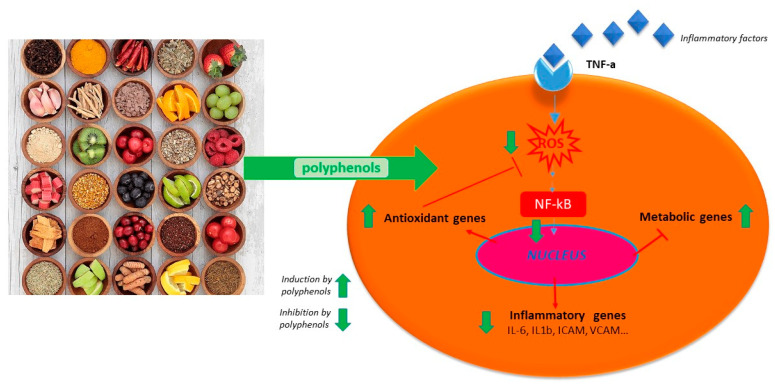
Schematic anti-inflammatory activities of natural polyphenols.

Polyphenols act on macrophages by inhibiting some of the key regulators of the inflammatory response such as TNF-α, IL-1-β, and IL-6 (Figure 1). A diet abundant in fruits rich in anthocyanins (such as red berry fruits) is related to a lower serum levels of IL-6, IL-12, and high sensitivity C reactive protein and therefore, to a decreased inflammation score in patient blood [65]. Moreover, polyphenol-enriched extra virgin olive oil and olive vegetation water have shown the capacity to reduce IL-6 and C-reactive protein expression. They also inhibit the production of TNF-α, which is usually activated by inflammation during a clinical trial on stable coronary heart disease patients and in vivo model system [59,64,66]. Table 1 shows the flavonoids able to inhibit the expression of pro-inflammatory cytokines like TNFα, IL-1β, IL-6, and IL-8, in several in vitro cell systems [43,54]. In addition, propolis extracts act as inhibitor on TNF-α, IL-1-β, and IL-6 in murine macrophages stimulated by LPS [67,68,69]. Likewise, polyphenols extract of chamomile, and isolated polyphenols such as quercetin from the extract, reduced the secretion of TNF-α and IL-6 without IL-1β modulation [60]. Polyphenols like quercetin and catechins work by balancing pro- and anti-inflammatory cytokines production; they enhance IL-10 release while inhibit TNFα and IL-1β [70] (Figure 2). A diet rich in such compounds may help patients with COVID-19 to reduce their inflammation due to the hyper-activation of cytokines such as TNFα, IL-1β, IL-6, and IL-8.

### 4.2. Active Anti-Inflammatory Plant Extracts 

In several studies, the biological activities have been evaluated by utilizing isolated single natural biomolecule. This approach was demonstrated to have both advantages and disadvantages. Advantages are a better understanding of a purified active molecule mechanism of action, and performing slight modifications on its structure to obtain molecules more efficacious (Table 1).

Polyphenols isolated from red wine, such as flavonols, soluble acids, and stilbenes such as trans-resveratrol were found to have anti-inflammatory effects [33]. Studies confirmed the anti-inflammatory activities of quercetin extracted from onions (*Allium cepa*), grape (*Vitis vinifera* L.), and red wine [33,58,71,72].

The anti-inflammatory activity of *Houttuynia cordata* Thunb., a perennial herbaceous plant mostly distributed in East Asia, has been studied [61]. Nowadays, fresh leaves of *H. cordata* are considered for a high-value industrial crop in Thailand and it has been fermented with probiotic bacteria to yield a fermentation product (HCFP) commercially available. The anti-inflammatory activities of aqueous and methanolic HCFP phenolic extracts have been demonstrated, and they are manifested by inhibiting the production of NO, PGE2, and inflammatory cytokines such as TNF-α, IL-1β, and IL-6, in in vitro and in vivo assays (see Table 1). 

Moreover, cannabinoids can suppress the immune activation and inflammatory cytokine production [73]. CB1 and CB2 are receptors for endocannabinoid. While CB1 is expressed overall in the central nervous system [33], CB2 is expressed by varieties of immune cells and in lymphoid tissues, airway tissues respond to both CB2 receptor-dependent and independent effects of cannabinoids [74,75,76]. Activation of CB2 receptor can suppress the release of inflammatory IL-1, IL-6, IL-12, and TNF-α [77].

Scientific data increasingly confirm the idea that the immunomodulatory approach targeting the over-production of cytokines could be proposed for viral pulmonary disease treatment. The peroxisome proliferator-activated receptor PPAR-γ, a member of the PPAR transcription factor family, also represses the inflammatory process and could represent a potential target [47]. In Table 1, we review the main nutritional PPAR-γ ligands, suggesting an approach based on the strengthening of the immune system exploiting dietary strategies as an attempt to contrast the rising of cytokine storm in the case of coronavirus infection. In addition, nutritional ligands of PPAR-like curcumin, carvacrol, carnosol, and capsaicin, possess anti-inflammatory properties through PPAR-activation.

### 4.3. Anti-Inflammatory and Anti-Viral Activity: The Case of Resveratrol

Among non flavonoid bioactive polyphenols, resveratrol has been studied for its capability to inhibit the growth of bacteria, fungi, viruses, and for its anti-inflammatory activity [78,79,80]. In particular, resveratrol inhibits virus-induced inflammatory mediators and the replication of several respiratory viruses such as rhinovirus, influenza A virus, respiratory syncytial virus, Middle East respiratory syndrome-coronavirus (MERS-CoV), and human meta pneumovirus [81,82,83,84,85]. It has been demonstrated that resveratrol could counteract the MERS-CoV-inducing apoptosis by down-regulating FGF-2 signaling [55]. Moreover, resveratrol may inhibit the NF-κB pathway activated by MERS-CoV, reducing inflammation [86,87]. Resveratrol could be used in patients with COVID-19 as well.

The therapeutic application of resveratrol as anti-inflammatory agent was delayed due to the low bioavailability. A recently published study reported the anti-inflammatory and anti-viral solution containing resveratrol plus carboxymethyl-β-glucan in children with allergic rhinitis and acute rhinopharyngitis [88,89]. The positive effects observed might be related to resveratrol and to the immune-modulation and osmotic activities provided by the glucan [90,91].

## 5. Conclusions 

The rapidly progressing SARS-CoV-2 pandemic has led to challenging decision-making about the treatment of critically unwell patients with COVID-19. This review demonstrates that, at least until the time of writing this paper, there is no proven cytokine blocking agents for COVID-19, the consciousness of the current “knowledge gap” can avoid premature favorable recommendations for potentially ineffective or harmful interventions. All human studies lack comparative data so that it remains unclear whether the patients recovered because of the use of a particular drug or the general clinical care received. Most in vitro studies, however, are suggestive of potential beneficial effects, although the data are preliminary, to be used as rationale for clinical use and clinical trials are ongoing. One of the major concerns is the toxicity and/or low efficacy of classical anti-inflammatory drugs. Here, some of the anti-inflammatory therapies currently under investigation have been discussed and novel therapies have been proposed. In particular, some IL-6 blocking agents are currently a matter of debate and more clinical trials are needed to better understand the feasibility of using such compounds in clinical practice. Other molecules such as JACK inhibitors seem, in some cases, associated to better outcome of pulmonary function. Indeed, all international guidelines recommend immunomodulatory drugs only in the context of clinical trials. Today, a strong suggestion is only for the use of systemic corticosteroid therapy in patients with severe and critical COVID-19. In the last decades, hundreds of research and review articles have been published regarding the anti-inflammatory activities of plants. Here, we provided evidence of the potential benefit of polyphenols in reducing the immune dysregulation. As described above, there is no known effective cure or treatment for patients with COVID-19 yet, thus all new potential treatment, or natural adjuvant-based strategies to be associated to classical strategies, could be able to reduce the severity of infection, and then are relevant and need to be investigated in detail. In this regards, bioactive phytochemicals, such as polyphenols may become promising tools for the treatment of COVID-19 in reducing the hyperactivation of cytokines such as TNFα, IL-1β, IL-6, and IL-8. Such nutrients with anti-inflammatory and antioxidant properties may prevent or attenuate the inflammatory and vascular manifestations associated with COVID-19. Plant bioactive molecules can be produced by renewable and low-cost source and this plays an important value for the enhancement and the exploitation of by-products of food chains. Moreover, following healthy dietary patterns may have beneficial effects to contrast infection, but require to be still explored. Further and more in-depth research about the topic discussed in this review is required in order to establish the effective strategies.

## Figures and Tables

**Table 1:** Natural polyphenols and their anti-inflammatory activity.

Plant Natural Products	Models of Inflammation	Main Effects on Inflammation	Ref.
Capsaicin, Carvacrol	Various in vitro and in vivo animal models (e.g., LPS-induced inflammation)	Inhibition of the production of TNF-α, activation of PPAR-α and γ and suppresses COX-2 expression	[46,47,48]
Lignans, Flavonoids	In-vitro studies in cells	Reduction in TNF-α and other cytokines levels; general–anti-inflammatory, analgesic and anti-allergic effects Analgesic effects and reduction of inflammatory measures	[32,49]
Curcumin, Apigenin, Quercetin, Cinnamaldehyde, Resveratrol Epigallocatechin-3-gallate	Various in vitro and in vivo animal models (e.g., LPS-induced inflammation) Adjuvant and carrageenan-induced arthritis in rats (acute and chronic designs) Various in vitro and in vivo models of inflammation; pre-clinical tests and clinical trials in humans	Reduction in IL-1β, IL-6, TNF-α, NO and PGs levels; inhibition of COX-2, iNOS and NFκB (nuclear factor kappa-light-chain-enhancer of activated B cells) and STAT3 (signal transducer and activator of transcription) activity Reduction of the expression of TLR-2 (toll-like receptor-2), PPARγ (Peroxisome proliferator-activated receptor gamma) Reduction in edema volume both in the acute and chronic models; effects comparable to those of phenylbutazone	[36,47,49,50,51,52,53]
Catethin, Epicatechin	In-vitro studies in cells LPS-induced inflammation in RAW 264.7 cells	Reduction of COX, IL-1β, TNF-α and IL-6 expression, and decrease of the activity of NF-κB Balance between pro- and anti-inflammatory cytokines production, enhancement IL-10 release, inhibition of TNFα and IL-1β	[34,35,54]
Carnosolic acid, Carnosol	Fusion receptor of the yeast Gal4-DNA binding domain joined to the hinge region and ligand binding domain of the human PPARγ in combination with a Gal4-driven luciferase reporter gene, cotransfected into Cos7 cells	Activation of Gal4-PPAR-γ fusion receptor, in a concentration-dependent manner.	[47,55]
Various polyphenols	LPS induced Inflammation in bone marrow derived dendritic cells	Reduction in IL-6, IL-12 and TNF-α levels	[56]
3-Hydroxyanthranilic acid	LPS-induced inflammation in RAW 264.7 and in peritoneal macrophages	Reduction of NO, IL-1β, IL-6 and TNF-α expression; a significant increase in IL-10 expression; reduction in NFκB activity	[57]
Curcumin analog EF31	LPS-induced inflammation in RAW 264.7 cells	Inhibition of the expression and secretion of TNF-α, IL-1-β, and IL-6	[58]
Anthocyanins	Humans	Reduction of inflammation score in patients’ blood, reflected by decreasing serum levels of IL-6, IL-12, and high sensitivity C reactive protein	[59]
Ferulic acid, Coumaric acid	LPS-induced inflammation in RAW 264.7 cells	Reduction of the production of TNF-α, interleukine-1-beta (IL-1-β) and IL-6 expression	[60]
Syringic, Vanillic, p-Hydroxybenzoic acids	LPS-induced inflammation in RAW 264.7 cells	Reduction of iNOS, COX-2, IL-1β, TNF-α, and IL-6	[61]
